# Practice patterns among thyroid cancer surgeons: implications of performing a prophylactic central neck dissection

**DOI:** 10.1186/s40463-016-0169-8

**Published:** 2016-10-28

**Authors:** Michael W. Deutschmann, Laura Chin-Lenn, Steven C. Nakoneshny, Joseph C. Dort, Janice L. Pasieka, Shamir P. Chandarana

**Affiliations:** 1 Division of Otolaryngology-Head & Neck Surgery, Department of Surgery, Red Deer Regional Hospital Center, Red Deer, Alberta Canada; 2 Division of General Surgery and Surgical Oncology, Department of Surgery, Cumming School of Medicine, University of Calgary, Calgary, Canada; 3 Ohlson Research Initiative, Arnie Charbonneau Cancer Institute, Cumming School of Medicine, University of Calgary, Calgary, Canada; 4 Division of Otolaryngology-Head and Neck Surgery and Surgical Oncology, Department of Surgery, Cumming School of Medicine, University of Calgary, Calgary, Canada; 5 Division of Endocrinology, Department of Medicine, University of Calgary, Calgary, Alberta Canada

**Keywords:** Thyroid cancer, Central neck dissection, Radioactive iodine, Epidemiology, Lymph node metastasis

## Abstract

**Background:**

Indications for performing a prophylactic central neck dissection (pCND) in papillary thyroid cancer (PTC) remain controversial. It is unclear how identification of lymph node (LN) metastases should impact the decision to treat with radioactive iodine (RAI). The goals of this study were to identify indications for performing pCND and identify factors that predict the use of adjuvant RAI.

**Methods:**

This was a population based cross-sectional analysis. A prospectively collected database identified 594 patients who underwent total thyroidectomy +/− CND. A multivariate model was constructed to identify indications for pCND and predictors of the use of RAI.

**Results:**

425 CNDs were performed of which 224 were prophylactic. Conventional risk factors (age, tumor size, extra-thyroidal extension) *were not* associated with performing a pCND. The presence of clinically suspicious lymphadenopathy was the only factor associated with performing CND, thus rendering the CND therapeutic. Positive LNs were retrieved in 39 % of pCND’s, upstaging 87 patients. Among all peri-operative predictors of receiving RAI, presence of LN metastases was the strongest predictor [OR = 5.9 (3.7–9.5)], while tumor size was a modest predictor [OR = 1.8 (1.5–2.1)]. Other conventional risk factors *did not* predict use of adjuvant RAI.

**Conclusions:**

Conventional risk factors were not indications for performing a pCND, implying that the decision was based on individual surgeon preference. Performing pCND upstaged 39 % of patients from cN0 to pN1a, increasing the likelihood of receiving RAI 6-fold. Conventional risk factors were not predictors of receiving adjuvant RAI. This highlights the need for a unified approach to performing a pCND and administering RAI.

## Background

Central neck dissection (CND) has an important, but controversial role in the treatment of papillary thyroid cancer (PTC). The central neck is the first nodal basin to which PTC spreads and when central neck nodes are affected, between 20 and 90 % of patients develop lateral neck compartment metastases [1, 2]. Therapeutic CND (tCND) in patients with clinically suspicious lymph node (LN) metastases is a well-established intervention. It is when patients have no clinically suspicious metastatic LNs that the role of prophylactic CND (pCND) becomes unclear. The American Thyroid Association (ATA) 2015 guidelines identify that the impact of pCND on survival is unclear, given that the survival in these patients is excellent overall [1, 3].

The 2009 ATA guideline recommended consideration of a pCND on those who have advanced primary tumors (T3 or T4), clinically involved lateral neck nodes (cN1b), or if the information will be used to plan further steps in therapy [1]. Following the publication of these guidelines, there was much debate in the surgical community as to the value of pCND in all or even in high risk PTC patients. Metastatic central compartment LN’s have been shown to be associated with risk factors such as gender, primary tumor size, *BRAF* mutations, primary extrathyroidal extension and evidence of lateral cervical LN metastases, leading some authors to utilize these features as indications for performing a pCND [4–6]. Small studies demonstrated that cervical LN metastases may affect overall survival in well differentiated thyroid cancer (WDTC) [7, 8]. In contrast, however, other large epidemiologic studies do not support the impact of performing a pCND on survival [9, 10]. A recent randomized controlled trial looking at the use of pCND in PTC showed undergoing pCND required fewer repeat doses of radioactive iodine (RAI), but significantly higher rates of permanent hypoparathyroidism [11]. The conflicting nature of the data led most surgeons to devise personal algorithms for when to perform a pCND, as well as the extent of the pCND (unilateral vs. bilateral CND).

Therefore, the objective of this study was to identify the pre-operative indications associated with performing a pCND among surgeons in the province of Alberta. We hypothesized that surgical decision-making was consistent with the ATA guidelines, with high-risk factors such as advanced age, larger primary tumor and evidence of extrathyroidal extension influencing a surgeon to perform a pCND. The second goal of this study was to determine predictors of receiving adjuvant RAI. We hypothesized that conventional risk factors would predict the use of RAI.

## Methods

Thyroid surgeons in the province of Alberta have the ability to record pre- and intra-operative data pertaining to patients undergoing thyroid surgery using a prospectively collected synoptic operative reporting system, known as the Alberta WebSMR. [12] The resulting data includes pre-operative parameters such as demographics, pre-operative staging, and evidence of any clinically suspicious LNs in the neck. It also contains peri-operative information such as size of the tumor, intra-operative findings, and intra-operative complications. This database was used to identify our patient cohort, and to provide pre-operative and peri-operative data.

All patients identified via the Alberta WebSMR database with a diagnosis of PTC who underwent, at minimum, completion or total thyroidectomy, with or without CND between January 1, 2009 and July 31, 2012 were included for analysis. Patient demographic data including gender and age were collected. Surgical data such as stage of cancer, type of surgery performed, extent of CND (unilateral vs. bilateral) and evidence of extrathyroidal extension were also collected. Pathology reports were reviewed to collect overall LN yield, number of pathologic LNs identified in the neck dissection sample, and final pathologic stage. The use and dosage of RAI was also recorded.

### Statistical analysis

Patient demographics, as well as pre-operative and intra-operative variables were compared between those who did and did not receive CND to determine associations between these factors and performing a CND. Categorical variables were compared using either a chi square or Fisher exact test as appropriate, while continuous variables were compared using either a Student’s *t*-test or Wilcoxon rank-sum test as appropriate. A *p*-value of less than or equal to 0.05 was deemed significant for all analyses.

With respect to identifying factors that predicted the use of RAI, a multivariable logistic regression model was constructed using ‘high-risk’ predictors (from the literature) as well as those predictors that met with statistical significance on univariate analysis. Odds ratios and confidence intervals for significant predictors were calculated. All final multivariable regression models were evaluated for goodness-of fit, model stability and influential observations.

Statistical analysis was performed using Stata (version 12.1, StataCorp LP, College Station, TX).

This study was reviewed and approved by the Alberta Cancer Research Ethics Committee.

## Results

Table 1 illustrates demographic and tumor characteristics, stratified by whether or not a patient underwent a CND. In total, 594 patients treated by 18 surgeons were included in our initial cohort. Of those, 425 (72 %) patients underwent CND. There were 313 unilateral and 112 bilateral CND with mean total LN yields of 7.4 +/− 6.3 nodes and 11.9 +/− 7.5 nodes respectively. Of the 425 patients that underwent CND, 224 (53 %) underwent a pCND and 201 (47 %) a tCND. In the 224 patients undergoing a pCND, none of the conventional risk factors, such as pathologic tumor size, age, or evidence of extrathyroidal extension were identified as factors that influenced the decision to perform a pCND. Overall, only the presence of pre-operative clinically suspicious LNs was associated with a CND (*p* < 0.0001), thus rendering the CND therapeutic.

**Table 1:** Clinical and pathologic patient factors stratified by whether patient received CND or not

	No CND	CND	*p* value
No. of patients	169	425	
Age (years)	46.3	45.6	ns
Sex			
Male	43	100	ns
Female	126	325	
Clinically suspicious lymph nodes in central neck			
Yes	6	81	<0.0001
No	105	224	
Procedure			
Total Thyroidectomy or Completion Thyroidectomy alone	169	0	
Total Thyroidectomy with Ipsilateral CND	0	313	
Total Thyroidectomy with Bilateral CND	0	112	
T stage			
T1a	32	79	ns
T1b	46	106	
T2	43	96	
T3	20	93	
T4a	2	14	
T4b	0	2	
M stage			
M0	157	414	ns
M1	0	2	
Tumor size (largest dimension in centimeters)	2.0	2.2	ns
Extrathyroidal Extension present	3	20	ns

*CND* central neck dissection, *ns* not significant

Three hundred twenty-nine patients were identified that had no preoperative suspicion of LNs in the central neck, of which 224 (68 %) underwent a pCND and 87 (39 %) of these patients were found to have at least one positive metastatic LN upon final pathologic evaluation. Adjuvant RAI was then administered to 55 % of these 87 patients. Table 2 demonstrates the results of the multivariable logistic regression model among patients who had a clinical N0 neck. On multivariate analysis, T stage was a modest predictor of receiving RAI [OR 1.83 (1.5–2.1)], while pathologic N Stage was the strongest predictor [OR 5.9 (3.7–9.5)]. The act of performing a pCND was found to be collinear with pathologic N stage, implying that performing a pCND increases the likelihood of receiving adjuvant RAI, due to the high chance of identification of LN metastases.

**Table 2:** Logistic regression: Factors that predict administration of adjuvant RAI

Predictor	OR	CI	*p*-value
Pathologic T stage	1.83	1.5–2.1	0.0001
Pathologic N stage	5.9	3.7–9.5	0.0001
Age	NS		
ETE	NS		

*RAI* radioactive iodine ablation, *ETE* extrathyroidal extension

When stratifying patients based on number of nodes retrieved, patients with greater than 5 positive nodes were the most likely to receive RAI [OR = 23.3 (8.3, 65.3)], followed by patients with 1 to 5 nodes identified [OR = 5.9 (3.8, 9.3)].

**Fig. 1:**
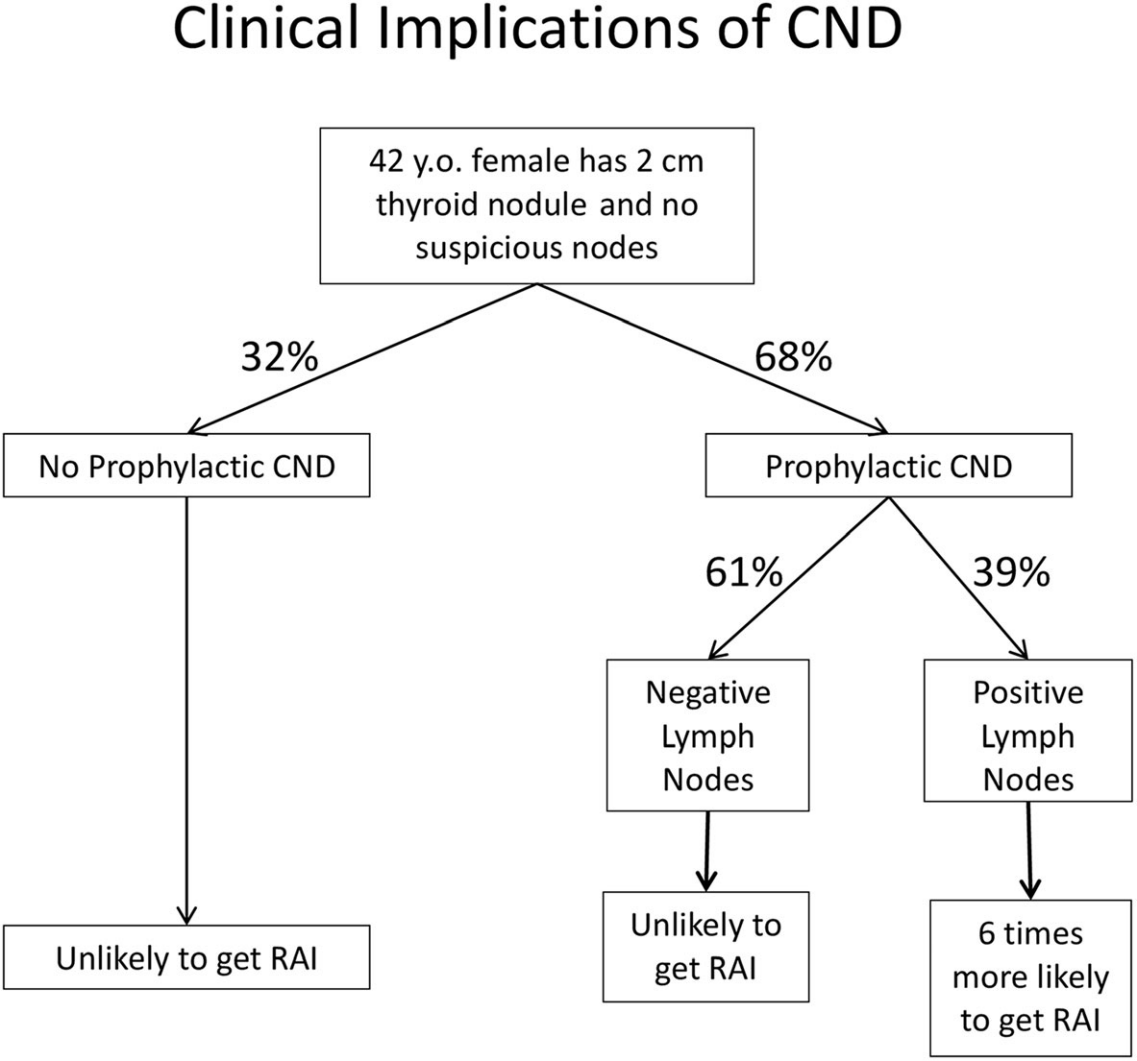
Potential impact of performing prophylactic CND on patient with low-risk well differentiated thyroid cancer

The flow chart in Fig. 1 demonstrates how a surgeon’s decision to perform a prophylactic CND affects a patient’s management in our cohort. In the province of Alberta, 68 % of patients without clinical evidence of LN metastasis received a pCND. After having performed the pCND, occult LN metastasis would be discovered in 39 %, thus upstaging the patient. As a result of having pathologically positive LNs, these patients are almost 6 times more likely to receive RAI.

## Discussion

The role of pCND has become a much-debated topic over the last several years. While there is little debate about how to manage clinically apparent central LN metastasis, the role of pCND is unclear [1–3]. A recent meta-analysis concluded that performing routine pCND increases the risk of temporary hypocalcaemia and does not improve loco-regional control [10]. This would argue that CND has some morbidity, but questionable efficacy. However, CND can provide prognostic information and influence the decision to administer RAI, leading some to advocate for more comprehensive staging and risk stratification [3, 13]. Current literature reflects that conventional risk factors such as age of patient, tumor size, extrathyroidal invasion, and lateral neck disease are associated with a higher likelihood of occult disease in the central LN’s, and as such are utilized as guidelines for performing a pCND [4–6].

In the initial analysis tumors were divided into six categories based on T-stage, and there was no correlation between tumor size and pCND. In order to ensure that our analysis was not limited by the fact that some sub-categories of T-stage had a limited sample size, a separate post-hoc analysis was performed by first dichotomizing patients into only two groups based on T – stage (T1-2 vs. T3-4). There was a non-significant trend toward increased likelihood of pCND in the T3-4 group. We also dichotomized patients based on age (age <45 vs. age >45) and again, found no association between dichotomized age and pCND.

In contrast to what is assumed to be the reason behind the adoption of pCND in Alberta following the publication of the ATA guidelines in 2009, we were unable to identify any conventional risk factors that influenced the decision to perform a pCND. Three-hundred-twenty-nine patients had no suspicious nodes pre-operatively, and in this subset, factors such as advanced tumor size, age and evidence of extrathyroidal extension failed to differentiate those who did receive a pCND from those who did not. This deviation from the ATA demonstrates that surgeons have developed a more surgeon-specific approach to performing a CND.

A recent study by the ATA showed that the number of pathologic LNs obtained in a CND may have more prognostic significance than simply the presence of any LN metastases [13]. This review found that prognosis changed only if 5 or more metastatic LNs were identified, emphasizing the need to perform a comprehensive CND. Previous work published by our group confirmed that comprehensive CNDs are being performed across our province [14]. It was the belief of the senior authors that by providing better staging material, patients who might otherwise have been candidates for adjuvant therapy in the past could now be observed.

However this study demonstrated that the decision to perform a pCND lead to a downstream decision to administer adjuvant therapy. The strongest predictor of receiving adjuvant RAI was the presence of LN metastasis (pN stage), while tumor size (pT stage) was only a modest predictor. In the province of Alberta, 68 % of patients without clinical evidence of LN metastasis received a pCND, whereby occult LN metastases were discovered in 39 %, thus significantly upstaging the patient. In patients with pathologically positive LNs, the likelihood of receiving RAI was almost 6 times higher. While the adverse effects of RAI are relatively low, the potential benefit from this intervention has not been clarified in the intermediate-risk patient population with a limited number of LN metastases. It is possible that a large number of these patients are being over-treated with adjuvant RAI [15].

The new 2015 guidelines suggest that patients are considered low risk if they are clinically N0 or have ≤5 pathologic lymph nodes with micrometastasis (<0.2 cm in largest dimension), whereas patients that are clinically N1 or have >5 pathologic lymph nodes are considered intermediate risk. Patients with intermediate level of risk may derive some benefit from RAI. In our cohort, patients with greater than 5 positive nodes were the most likely group to receive RAI. Despite the fact that our cohort was assembled prior to the release of these updated guidelines, it would appear we are compliant with the current risk stratification. With further improvements in compliance to this proposed risk stratification, future patients may either be appropriately spared adjuvant RAI, or may more clearly warrant the use of adjuvant RAI, depending on risk level.

One limitation of this study is the variable compliance among surgeons in using the Alberta WebSMR synoptic reporting system. It is not mandatory for surgeons to utilize this database, thus, our data represents the majority, rather than the entirety, of thyroid procedures carried out in the province. While it is possible that some surgeons may be selective in which patients they enter into the database, we believe that for those surgeons who do enter patients into the database, all consecutive patients are entered.

Although data for this study was collected over a short 3-year period, during this time endocrinologists in our province began to use baseline, post-operative stimulated thyroglobulin and low dose I131 whole body scans prior to administration of therapeutic doses of RAI. Therefore, it was not feasible to look at baseline stimulated thyroglobulin and whole body scans as factors that may predict the use of RAI in our cohort. It is therefore possible that there were other factors that influenced the administration of RAI then was analyzed.

This study is unique in that we were able to assess practice patterns among surgeons from different institutions and surgical disciplines. The homogeneity of the large sample size selected from the provincial population base of 3.6 million enables us to generalize our findings beyond institutional borders and renders the evaluation of a large number of independent predictors feasible. This study highlights the lack of consensus across the province both with respect to indications for performing a pCND as well as for receiving adjuvant RAI. Although the ATA guidelines are frequently referred to in addressing practice management, specialists in our province were not uniform in adherence. Rather, specialists appeared to develop individual, practice-based treatment algorithms as it relates to performing a pCND and administering RAI. With the recent publication of the 2015 ATA guidelines, refinement of the indications for pCND, risk stratification and utilization of RAI administration will need to be addressed within our provincial group of specialists. While there is often a lag time in guideline adherence, with the results from this study, our group is now ideally situated to institute an intervention that would improve consistency among specialists, and thus standardize treatment across the province.

## Conclusions

Indications for performing a pCND appear to vary among surgeons across the province, and were not always consistent with conventional risk factors for high risk PTC. Currently, the only consistent factor that influences the decision to perform a CND is the presence of clinically detected suspicious lymphadenopathy. In the setting where no pre-operative suspicious nodes were apparent, 39 % of patients who underwent pCND had their nodal status upstaged and in these patients, the likelihood of receiving adjuvant RAI was increased 6-fold because of the identification of one or more metastatic LNs.
